# Hypomethylating Agents and Immunotherapy: Therapeutic Synergism in Acute Myeloid Leukemia and Myelodysplastic Syndromes

**DOI:** 10.3389/fonc.2021.624742

**Published:** 2021-02-25

**Authors:** Kah Keng Wong, Rosline Hassan, Nik Soriani Yaacob

**Affiliations:** ^1^ Department of Immunology, School of Medical Sciences, Universiti Sains Malaysia, Kelantan, Malaysia; ^2^ Department of Haematology, School of Medical Sciences, Universiti Sains Malaysia, Kelantan, Malaysia; ^3^ Department of Chemical Pathology, School of Medical Sciences, Universiti Sains Malaysia, Kelantan, Malaysia

**Keywords:** acute myeloid leukemia, myelodysplastic syndromes, hypomethylating agents, cancer vaccine, immune checkpoint, chimeric antigen receptor-engineered (CAR)-T cell therapy

## Abstract

Decitabine and guadecitabine are hypomethylating agents (HMAs) that exert inhibitory effects against cancer cells. This includes stimulation of anti-tumor immunity in acute myeloid leukemia (AML) and myelodysplastic syndromes (MDS) patients. Treatment of AML and MDS patients with the HMAs confers upregulation of cancer/testis antigens (CTAs) expression including the highly immunogenic CTA NY-ESO-1. This leads to activation of CD4^+^ and CD8^+^ T cells for elimination of cancer cells, and it establishes the feasibility to combine cancer vaccine with HMAs to enhance vaccine immunogenicity. Moreover, decitabine and guadecitabine induce the expression of immune checkpoint molecules in AML cells. In this review, the accumulating knowledge on the immunopotentiating properties of decitabine and guadecitabine in AML and MDS patients are presented and discussed. In summary, combination of decitabine or guadecitabine with NY-ESO-1 vaccine enhances vaccine immunogenicity in AML patients. T cells from AML patients stimulated with dendritic cell (DC)/AML fusion vaccine and guadecitabine display increased capacity to lyse AML cells. Moreover, decitabine enhances NK cell-mediated cytotoxicity or CD123-specific chimeric antigen receptor-engineered T cells antileukemic activities against AML. Furthermore, combination of either HMAs with immune checkpoint blockade (ICB) therapy may circumvent their resistance. Finally, clinical trials of either HMAs combined with cancer vaccines, NK cell infusion or ICB therapy in relapsed/refractory AML and high-risk MDS patients are currently underway, highlighting the promising efficacy of HMAs and immunotherapy synergy against these malignancies.

## Introduction

Acute myeloid leukemia (AML) and myelodysplastic syndromes (MDS) are clonal stem cell disorders characterized by heterogeneous clinical outcomes due to underlying molecular and cytogenetic architectures (1, 2). Chemotherapy regimen with or without allogeneic hematopoietic stem cell transplantation (allo-HSCT) is a main therapeutic option for AML patients. The “7+3” regimen (7 days of cytarabine and 3 days of an anthracycline antibiotic or an anthracenedione) is a first-line induction chemotherapy for AML patients who could tolerate intensive therapy. However, older AML patients aged ≥60 or therapy-related AML receiving 7 + 3 regimen demonstrate poor complete response (CR) rates of <50% (3–5). Moreover, relapsed or refractory (R/R) AML patients unfit for induction chemotherapy demonstrate poor median overall survival (OS) of merely a few months (6, 7). In addition, allo-HSCT confers the risk of graft-versus-host disease (GvHD) that remains lethal. High-risk MDS has limited treatment options and patients are often ineligible for intensive chemotherapy caused by comorbidities or complex biological features that often result in chemotherapy resistance (8).

Immunotherapy has revolutionized cancer treatment where it harnesses and activates patient’s own immune system to destroy cancer cells. Immunotherapy is termed as the “fifth pillar” of cancer therapy that complements or supersedes conventional first-line cancer therapy. However, progress in the application of immunotherapy in AML and MDS such as antibody-based therapy, cellular immunotherapy, immune checkpoint blockade (ICB) agents and cancer vaccines has been slower compared with solid tumors (9, 10). Currently, only gemtuzumab ozogamicin, an anti-CD33 antibody-drug conjugate, has been approved as an antibody-targeted therapy for CD33-positive AML patients (10, 11). Phase I and II clinical trials have assessed ICB therapy based on programmed death-1 (PD-1) and cytotoxic T-lymphocyte-associated protein 4 (CTLA-4) inhibition but they have yielded modest clinical efficacy (12). These underscore the unmet need to enhance the efficacy of immunotherapy with other agents for AML and MDS treatments.

Epigenetics regulation is inherited DNA modifications and external alterations of the physical DNA structure that affect gene expression profiles without modifying nucleotide sequence. The epigenetics modifications include DNA methylation, hydroxymethylation, post-translational histone modifications and nucleosome remodeling (13–15). DNA methylation occurs predominantly at cytosine residues specifically at the carbon-5 position of cytidine found in promoter CpG islands, resulting in 5-methylcytosine (5mC) typically associated with transcriptional repression (16, 17), affecting a variety of cellular processes. DNA methylation is mediated by canonical DNA methyltransferases (DNMTs) consisting of DNMT1, DNMT3A and DNMT3B that facilitate the methylation of genomic DNA (18). Canonical DNMTs have been frequently implicated as oncoproteins in multiple tumor types where they promote tumorigenesis, cancer cell cycle progression, proliferation and immune escape in both solid (19–23) and blood cancers (24–26) including AML (27–30).

**Figure 1:**
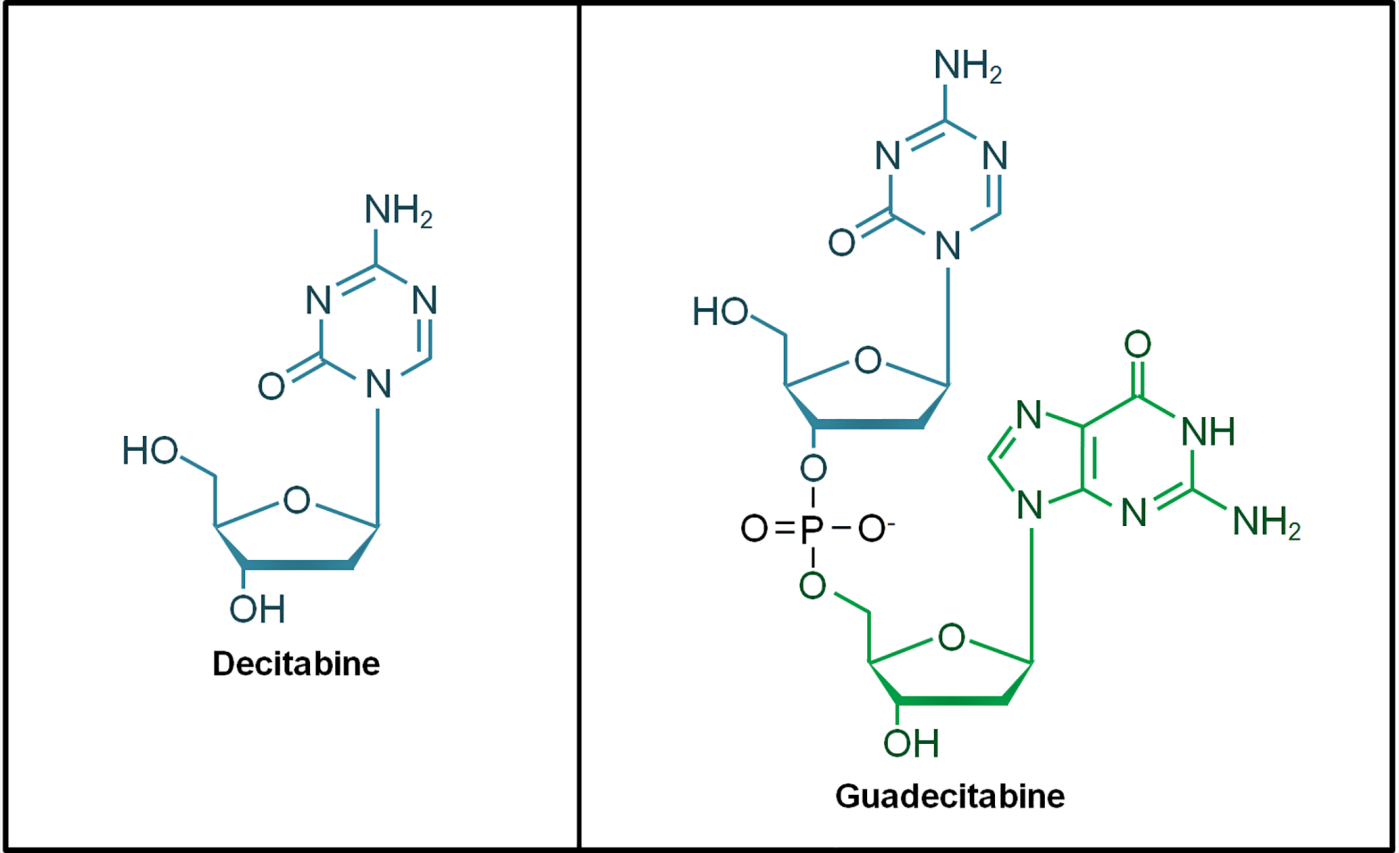
Chemical structure of decitabine and guadecitabine. Guadecitabine is a dinucleotide of decitabine (in blue) and deoxyguanosine (in green) joined by a phosphodiester bond.

The hypomethylating agents (HMAs) 5-azacytidine (azacitidine) and 5-aza-2’-deoxycytidine (decitabine) are cytidine nucleoside analogs approved for the treatment of AML and MDS patients. These HMAs mimic cytosine to incorporate into DNA during cellular replication, forming a scaffold that traps DNMTs for proteasomal degradation. This leads to DNA hypomethylation that restores gene transcription particularly tumor suppressor genes in AML (31, 32). Guadecitabine (SGI-110) is a second-generation HMA and it is a dinucleotide of decitabine and deoxyguanosine (**Figure 1**). Guadecitabine has been developed to address the shortcomings of first-generation HMAs that are susceptible to deamination by cytidine-deaminase (CDA) found in multiple organs in the body, leading to their short plasma half-life. Guadecitabine is more resistant to CDA with improved stability that confers enhanced DNA incorporation into dividing cells (33–35).

A growing body of evidence has demonstrated the potential of HMAs to promote the efficacy of immunotherapy in AML and MDS patients. In this review, the immunopotentiating effects of decitabine and its dinucleotide guadecitabine to sensitize AML and MDS patients for immunotherapy are presented and discussed. The immunomodulatory properties of azacitidine in AML or MDS patients have been documented in experimental or clinical settings (36–38), and its combination with various immunotherapeutic modalities have shown promises in the induction of antileukemic T or NK cells immunity in early phase clinical trials of AML (39) or MDS (40) patients. Nonetheless, its lack of efficacy in eradicating leukemic stem cell (LSC) or progenitor cell populations in AML and MDS patients have been noted (41, 42). For extended information of azacitidine combination with immunotherapy for the treatment of AML and MDS patients, readers are directed to a recent review by Daver et al. (43).

## Activation of Antileukemic T Cells

### Upregulation of Cancer/Testis Antigens (CTAs)

CTAs are a group of tumor-associated antigens (TAAs) whose expression profile is absent in normal adult tissues but highly expressed in normal testicular germ cells and placenta trophoblasts, as well as various types of cancers (44–46). CTAs participate in diverse neoplastic processes such as transcriptional regulation, mitotic fidelity and protein degradation that collectively antagonize tumor-suppressive mechanisms (47). CTAs were originally identified in the early 1990s by T cell epitope cloning as targets of cytotoxic T cells (CTLs) such as MAGE-A, BAGE, and GAGE-A. The SEREX (serologic identification of antigens by recombinant expression cloning) methodology, which is capable of isolating TAAs that elicit high titers of human immunoglobulin G (IgG), has since expanded the list of CTAs including NY-ESO-1, CAGE, and SSX-2. In particular, NY-ESO-1 is one of the most well-studied CTAs whereby its expression in cancers elicits strong and specific anti-tumor responses. CTAs expression outside of their naturally-occurring immune-privileged sites could trigger immune responses (47). Epigenetics modifications including DNA demethylation and histone modifications have been implicated in the overexpression of CTAs leading to tumor immunogenicity (48, 49). Treatment with HMAs has been shown to induce CTAs expression that triggers CTL antileukemic responses in AML and MDS as described in the next paragraphs.

Expression of CTAs is often very low or absent in myeloid leukemias due to hypermethylation of the gene promoters. In mouse leukemia cells (L1210), the expression of P1A, a mouse CTA, was upregulated by decitabine treatment. P1A-specific CTLs generated from decitabine-treated DBA/2 mice showed markedly increased cytotoxicity against leukemia cells (L1210) treated with decitabine, indicating that the compound could induce the production of autologous CTA-specific CTLs *in vivo* against leukemia cells (50). Treatment of multiple human acute leukemia cell lines (Kasumi-1, U937, NB4, THP-1, Jurkat, and Molt-4) with decitabine activated the expression of the CTA nuclear RNA export factor 2 (*NXF2*). Bone marrow samples derived from primary acute leukemia patients (n=8) also showed upregulation of *NXF2* mRNA expression following decitabine treatment, and *NFX2* was also upregulated in all AML or MDS patients (n=9) treated with decitabine (51). Consistent with the hypomethylating properties of decitabine, the increased expression of *NXF2* mRNA expression was associated with demethylation of its promoter region CpG islands in leukemia cells (K562 and U937). However, CTL responses against NXF2-positive AML cells following decitabine treatment was not demonstrated in the study due to lack of known epitope sequence of NXF2 when the study was conducted.

Another CTA termed as preferentially expressed antigen in melanoma (PRAME) whose expression is primarily upregulated by DNA demethylation and its expression has been associated with favorable outcomes in leukemias including AML (52). This suggests that PRAME is an ideal immunotherapy target when its expression is restored therapeutically. PRAME expression can be enhanced by decitabine treatment in combination with an histone deacetylase inhibitor (HDACi) chidamide in AML cells. Pre-treatment of HLA-A*0201^+^ AML cells (THP-1) with chidamide and/or decitabine induced sensitivity to CTLs that recognized PRAME peptides presented by HLA-A*0201 on AML cells, and susceptible to cytotoxicity by PRAME-specific CTLs (53). However, pre-treatment with chidamide alone (but not decitabine) inhibited proliferation of activated CD4^+^ and CD8^+^ T cells. Moreover, as noted by the authors, it was unclear if chidamide treatment may stimulate PRAME expression in other normal tissues apart from AML cells. These suggest that alternative HDACi in combination with decitabine might be more efficient in conferring higher and more specific anti-tumor CTL responses against AML cells.

Decitabine treatment also augmented the CTAs MAGE-A1, MAGE-A3 and SP17 expression in MDS (SKM-1) and chronic myeloid leukemia (CML) (K562) cell lines. In MDS patient samples, the compound increased CTA-specific CTL recognition of upregulated CTAs in bone marrow cells of MDS patients, along with enhanced CTL function and increased expression of major histocompatibility complex (MHC) class I and II proteins as well as ICAM-1 (a cell adhesion molecule that enhances binding with T cells for tumor lysis) (54). Nonetheless, low levels of cytotoxicity against partially HLA-matched leukemia cell lines (SKM-1 and K562) by tumor-specific CTLs (derived from MDS patients treated with decitabine) were observed in the same study. The low-level cytotoxicity may be due to partial matching of HLA haplotypes, and it was unclear if prior exposure to chemotherapy also played a contributive role. Chemotherapy-induced augmentation of inhibitory surface receptors such as PD-1 on T cells leading to exhaustion has been reported in chronic lymphocytic leukemia (55). However in AML patients, increased expression of inhibitory receptors such as PD-1 and TIM3 have only been observed in relapsed or patients unresponsive to chemotherapy (56), and increased frequencies of PD-1^+^TIGIT^+^CD226^−^CD8^+^ T cells were associated with failure to achieve remission after induction chemotherapy (57).

Guadecitabine treatment conferred overexpression of CTAs NY-ESO-1 and MAGE-A through promoter hypomethylation in leukemia cells *in vitro* (HL60, U937 and KG1a), and in AML xenografts *in vivo* (U937 in SCID mice). The CTAs upregulation induced cytotoxicity by HLA-compatible CTLs specific for NY-ESO-1 with increased expression of pro-inflammatory cytokines (*e.g.* IFN-γ and TNF-α) by the CTLs. This might be achieved through upregulation of MHC class I and expression of co-stimulatory molecules required for CTAs presentation. Essentially, guadecitabine at near-equivalent molar doses as decitabine was as efficient as decitabine in promoting CTA and co-stimulatory molecules expression (58).

In human AML cells (Kasumi-1), treatment of decitabine induced the transcript expression of numerous CTA genes preferentially located on the X-chromosome including *NY-ESO-1*, *MAGEA3*, *MAGEB2*, *GAGE 1-4*, *SSX 1-4*, and *XAGE1* where each of these genes showed at least 5-fold induction after 3 days of treatment (59). *NY-ESO-1* demonstrated the biggest fold induction of over 200-fold 3 days post-treatment, and over 250-fold 6 days post-treatment. Moreover, decitabine-induced NY-ESO-1 protein expression in AML cells (U937) elicited a specific and time-dependent CTL responses *in vitro*, and that *de novo* expressed NY-ESO-1 protein was effectively processed and presented by day 6 post-treatment with decitabine. The authors also further showed that CTL responses were cytotoxic against decitabine-treated AML cells (U937), NY-ESO-1 positive AML cells (U937 pulsed with NY-ESO-1 p94-102 peptide), and HLA-B51/NY-ESO-1 positive melanoma cells (LB39) but without cytotoxicity against NY-ESO-1 negative cells (MZ-Mel-7) *in vitro*. These cell line studies suggest that NY-ESO-1-specific CTLs may be induced by decitabine treatment in AML patients.

Indeed, peripheral blood blasts of AML patients receiving standard decitabine monotherapy (20 mg/m^2^ daily for 10 days) showed increased *NY-ESO-1* and *MAGEA3/A6* expression regardless of clinical response, along with promoter-specific and global (*LINE-1*) hypomethylation. Critically, AML blasts isolated from HLA-A*0201^+^ AML patients treated with decitabine sufficiently stimulated HLA-A*0201-restricted NY-ESO-1-specific CTL responses as shown by increased levels of intracellular cytokines in HLA-A*0201/NY-ESO-1_157-165_ tetramer^+^ CD8^+^ T cells (60). Hence, decitabine treatment resulted in AML cells expressing NY-ESO-1 at levels sufficient to elicit recognition by NY-ESO-1-specific T cells. This suggests that vaccination against NY-ESO-1 combined with decitabine monotherapy might be effective to treat AML patients.

In a clinical study, a conditioning strategy with decitabine regimen was designed for low toxicity with hopes to achieve sufficient myelosuppressive activity and immune-enhancing effects in high-risk MDS, AML and chronic myelomonocytic leukemia (CMML) patients (n=30 in total). The standard decitabine regimen (20 mg/m^2^/day for 10 days) was combined with fludarabine and low-dose total body irradiation (TBI; 2 Gray) regimen (Dec/Flu/TBI). The Dec/Flu/TBI regimen demonstrated tolerable response with promising OS (53%) post-HSCT. Immunomonitoring showed that CTA-reactive (MAGE-A1/A2/A3 and PRAME) CTL responses post-HCT occurred more frequently in patients who received Dec/Flu/TBI (n=8/11; 72.7%) than those with only Flu/TBI conditioning (n=2/9; 22.2%), indicating that decitabine increased CTA-specific T cell responses for improved responses in clinical settings (61). Essentially, combination of the conditioning regimen with decitabine did not result in increased incidence or severity of GvHD that may result from augmented expression of CTAs in healthy tissues, suggesting a leukemia-specific increase in CTA expression by decitabine. Hence, decitabine could serve as an adjunct to vaccination against CTAs in post-HSCT AML patients.

### Cancer Vaccine

Targeting of surface receptors of dendritic cells (DCs) with antibodies can lead to increased immunogenicity. DCs are frequently adopted in cellular vaccine clinical trials due to their essential roles in presenting antigens to activate immune responses, and capable of conferring promising clinical responses in advanced cancers including AML shown in early phase clinical trials (62). A common DC receptor targeted through this approach is deca-lectin DEC-205 (CD205) that mediates antigen uptake and presentation (63). The fully human anti-DEC-205 monoclonal antibody (mAb) is fused to the full-length NY-ESO-1 antigen (the anti-DEC-205-NY-ESO-1 fusion protein is also known as CDX-1401) and it is a DC-targeted antibody vaccine for presentation of NY-ESO-1 antigen to activate T cells. Combination of anti-DEC-205-NY-ESO-1 fusion protein with poly-ICLC (polyinosinic-polycytidylic acid and poly-L-lysine, a synthetic dsRNA complex that acts as a viral mimic recognized by the endosomal receptor TLR3) enhances NY-ESO-1 antigen presentation for T cell immune responses (64). As NY-ESO-1 expression in leukemia cells has been consistently shown to be increased by decitabine treatment in pre-clinical settings, this property forms another strategy to further enhance NY-ESO-1 presentation by DC vaccines to treat leukemia patients.

In a phase I study (NCT01834248), MDS patients receiving HLA-unrestricted NY-ESO-1 vaccine (anti-DEC-205-NY-ESO-1 fusion protein combined with poly-ICLC) every 4 weeks with decitabine at standard dose (20 mg/m^2^/day) showed increased NY-ESO-1 expression in all seven patients investigated. NY-ESO-1-specific CD4^+^ and CD8^+^ T cell responses were achieved in 85.7% (n=6/7) and 57.1% (n=4/7) of the vaccinated patients, respectively. Moreover, NY-ESO-1^+^ myeloid cells from one of the patients on decitabine therapy activated cytotoxic responses from autologous NY-ESO-1-specific T cells (65). CD141^hi^ conventional DCs (cDCs) express higher levels of DEC-205 and TLR3 than CD1c^+^ cDCs. In normal immune processes, CD1c^+^ cDCs promote Th2 and Th17 immune responses against extracellular pathogens and CD4^+^ T cell priming, while CD141^+^ cDCs induce Th1 immune responses with a role in CD8^+^ T cell priming against tumor cells (66). Increased frequency of CD141^hi^ cDCs at diagnosis was associated with NY-ESO-1-specific immune responses, and that two MDS patients with higher CD141^hi^ cDCs post-treatment showed a prolonged clinical response to decitabine (65). It was unclear in the study if CD1c^+^ cDCs also contributed to NY-ESO-1 antigen presentation in the patients receiving the NY-ESO-1 vaccine. Nevertheless, the study showed for the first time that vaccination against NY-ESO-1 was safe and that the vaccine was capable of capitalizing on decitabine-mediated induction of NY-ESO-1 expression in malignant myeloid cells to enhance NY-ESO-1-specific, MDS-directed CTL immune responses. The clinical trials involving decitabine combination with cancer vaccine or other immunotherapy regimen in AML and MDS patients are summarized in **Table 1**.

**Table 1:** Clinical trials of decitabine combined with cancer vaccine or other immunotherapy regimen in AML and MDS patients.

Treatment	Phase & ID	Patients	Primary & secondary outcome measures	Primary completion
Decitabine + NY-ESO-1 vaccine (anti-DEC-205-NY-ESO-1 fusion protein and poly-ICLC)	Phase I; NCT01834248	MDS (n=7 patients reached the end-of-study)	Primary: Safety; Secondary: Change in immune and molecular epigenetic response	Completed in March 2016
Decitabine + NY-ESO-1 vaccine (anti-DEC-205-NY-ESO-1 fusion protein and poly-ICLC) + Nivolumab (anti-PD-1 mAb)	Phase I; NCT03358719	AML and MDS (n=8)	Primary: Safety; Secondary: Immune cell profile, and peripheral blood and BM cells responses (NY-ESO-1 expression and methylation)	December 2020
Decitabine + DLI + Autologous DC vaccine (pulsed with MAGE-A1, MAGEA3, NY-ESO-1)	Phase I; NCT01483274	Relapsed AML post-HSCT (n=N/A)	Primary: Safety; Secondary: Disease responses, and T cell responses to the CTAs	Withdrawn (adult patient population barriers)
Decitabine + Donor NK cells + Aldesleukin (recombinant IL-2)	Phase I; NCT02316964	R/R AML (n=8)	Primary: Safety; Secondary: Responses, detection of infused NK cells	Completed in December 2019
Decitabine + Cytarabine + All-transretinoic acid + G-CSF (DLAAG)	Phase II; NCT03356080	R/R AML and MDS with blast excess (n=50)	Primary: Safety; Secondary: Survival, adverse reactions, duration of hospitalization, rate of relapse	July 2020

BM, Bone marrow; DLI, Donor lymphocyte infusion; G-CSF, Granulocyte colony-stimulating factor; R/R, Relapsed or refractory.

Treatment of AML cells (THP-1) with guadecitabine resulted in increased expression of HLA-A2.1 with increased antigen presentation. *In vivo* model (mouse AML cells TIB-49 in C57BL/6J mice) treated with guadecitabine displayed T cells with reduced PD-1 levels but increased IFN-γ expression, as well as decrease in myeloid-derived suppressor cell (MDSC) populations (67). Personalized cancer vaccine was previously developed by the same research group whereby patient-derived AML cells fused with autologous DCs produced hybridoma (*i.e.* DC/AML fusion vaccine) successfully stimulated anti-tumor responses *via* the expansion and infiltration of leukemia-specific T cells, leading to prolonged remissions in AML patients post-chemotherapy (68). In their subsequent studies, T cells from AML patients stimulated with the DC/AML vaccine showed increased capacity to lyse AML cells when pre-treated with guadecitabine. Moreover, the DC/AML vaccine combined with guadecitabine treatment induced leukemia-specific immunity in an immunocompetent murine leukemia model (TIB-49 in C57BL/6J mice) (67). Interestingly, T cells from these mice demonstrated reduced expression of PD-1 upon guadecitabine treatment and addition of the vaccine did not further change PD-1 levels, in contrast with upregulated expression of PD-1 by decitabine treatment as described earlier. Possible explanations might be due to only one cycle of guadecitabine therapy as conducted in this study was not sufficient to induce *PDCD1* gene (that encodes PD-1 protein) promoter demethylation.

### Immune Checkpoint Blockade Therapy

The co-inhibitory receptor PD-1 and programmed death ligand-1 (PD-L1) are key immune suppressive factors whereby activation of PD-1 by its ligand PD-L1 induces immune tolerance (69–71). CTLA-4 is another co-inhibitory receptor commonly expressed on T cells where binding to its ligands CD80 and CD86 results in T cell inhibition (72, 73). In malignancies, the PD-1/PD-L1 axis or CTLA-4 signaling is exploited by cancer cells for immune escape (74–77). Targeting PD-1/PD-L1 axis and CTLA-4 with ICB against PD-1 (*e.g.* nivolumab, pembrolizumab), PD-L1 (*e.g.* atezolizumab, durvalumab) and CTLA-4 (*e.g.* ipilimumab) has revolutionized the therapeutic landscape of solid cancers. However, early phase I and II clinical trials of ICB therapy in AML patients have shown limited success and ICB has not been approved for treatment of AML patients. This is due to low immunogenicity of AML compared with solid tumors such as melanoma and NSCLC that are more immunogenic due to higher mutation rates (78). Cancer cells with higher mutation rates demonstrate increased odds of presenting antigens recognized by T cells as non-self (79), resulting in better response to immunotherapy (80–82). Furthermore, the bone marrow niche where AML and MDS tumor cells reside has a protective immunosuppressive microenvironment (83, 84).

In mice model infected with lymphocytic choriomeningitis virus (LCMV), decitabine treatment followed by blockade of PD-1 reinvigorated the function of exhausted gp33-specific (a dominant LCMV epitope) CTLs. In particular, sequential decitabine treatment and PD-1 blockade induced the proliferation of virus-specific CTLs, suggesting that exhaustion-associated *de novo* methylation programs were reversed by decitabine-mediated DNA hypomethylation, leading to enhanced CTLs expansion during ICB therapy (85). In human AML cell lines (KG-1 and THP1), expression of *PD-L1*, *PD-L2*, *PD-1* and *CTLA-4* transcripts was upregulated by decitabine treatment in a dose-dependent manner. PD-L1 and PD-1 protein expression was increased by decitabine treatment at concentration as low as 0.1 µM and also in a dose-dependent manner. In line with this, demethylation of *PD-1* CpG island loci was induced by decitabine treatment in AML cells (KG-1) (86). An independent study also showed that both decitabine and guadecitabine increased PD-1, PD-L1, and CTLA-4 expression in a panel of eight hematological cancer cell lines including AML (87). These findings suggest that resistance to HMAs in AML and MDS patients may be due to upregulated expression of immune checkpoint molecules, leading to exhaustion of CTLs and incomplete clearance of leukemia cells. Hence, ICB therapy may circumvent resistance to HMAs.

However, the effect of decitabine on immune checkpoint receptors expression in actual clinical settings is unclear. Donor lymphocyte infusion (DLI) for relapsed AML patients following HSCT is not particularly effective where the overall remission rates are between 15%–42% and with low OS of approximately 15%–20% (88–90). Moreover, a second HSCT in these patients demonstrate low long-term survival of only 10%–35% and with high treatment-related mortality of 50% (90). Priming of patients with azacitidine before DLI confers improved remission rates in relapsed AML and MDS patients (91–93). In retrospective studies, combination of decitabine with DLI in these patients has also been investigated where decitabine confers clinical efficacy in relapsed AML or MDS patients post-HSCT including patients with previous azacitidine failure (94, 95) and not restricted to patients demonstrating low leukemic burden (96). However, in bone marrow myeloblasts derived from relapsed AML or MDS patients post-HSCT (n=4), PD-L1 protein expression did not change considerably after treatment with decitabine and DLI (96). The number of patients investigated in the retrospective study was small and the relapsed patients were heavily pre-treated at HSCT and before decitabine treatment. A recent phase I trial (NCT02996474) that attempted to exploit on the graft-versus-leukemia (GvL) effect, combination of decitabine with pembrolizumab was administered in R/R AML patients (n=10) that showed tolerable safety with a toxicity profile largely comparable with that of decitabine monotherapy. Four of the patients (40%) demonstrated stable disease and one patient (10%) achieved an minimal residual disease (MRD)-negative CR at the end of the eight cycles (24 weeks) of therapy (97). The trial showed the feasibility of decitabine and pembrolizumab combination therapy in R/R adult AML patients, nevertheless it remains to be determined if decitabine could upregulate the expression of immune checkpoint receptors for effective ICB therapy and GvL in R/R AML patients post-HSCT. The clinical trials involving decitabine combination with ICB therapy in AML and MDS patients are summarized in **Table 2**.

**Table 2:** Clinical trials of decitabine or guadecitabine combined with ICB immunotherapy in AML and MDS patients.

Treatment	Phase & ID	Patients	Primary & secondary outcome measures	Primary completion
Arm 1: Decitabine + PDR001 (anti-PD-1 mAb) Arm 2: Decitabine + MBG453 (anti-TIM-3 mAb) Arm 3: Decitabine + PDR001 + MBG453	Phase: Ib; NCT03066648	R/R or *de novo* AML and high-risk MDS (n=235)	Primary: Safety, tolerability of MBG453; Secondary: AUC, C_max_, T_max_, half-life, response rates, survival, time to progression	April 2021
Decitabine + Pembrolizumab (anti-PD-1 mAb)	Phase: Ib; NCT03969446	R/R or new AML and MDS (n=54)	Primary: Safety, CR and CRi; Secondary: Response duration, survival and immunomonitoring (PD-1, PD-L1, and PD-L2 levels, T cell subsets)	June 2021
Decitabine + Venetoclax (BCL2 inhibitor) + Nivolumab (anti-PD-1 mAb)	Phase I; NCT04277442	AML (n=13)	Primary: Safety and responses; Secondary: Survival, MRD, T cell response, DNA methylation (global and specific immune checkpoint genes)	February 2022
Decitabine + Pembrolizumab (anti-PD-1 mAb)	Phase I; NCT02996474	R/R AML (n=10)	Primary: Safety; Secondary: Efficacy	Completed in April 2019
Guadecitabine + Atezolizumab (anti-PD-L1 mAb)	Phase Ib; NCT02892318	Cohort A1: Safety cohort (R/R AML; n=9); Cohort A2: Expansion cohort (R/R AML; n=11); Cohort A3: Safety cohort (untreated AML; n=6); Cohort A4: Expansion cohort (untreated AML; n=14)	Primary: Safety, CR, CRp, CRi, duration of response; Secondary: Survival, MRD, drugs concentration	December 2019*
Decitabine + Ipilimumab (anti-CTLA mAb)	Phase I; NCT02890329	R/R AML and MDS (post allo-HSCT patients or transplant-naive patients; n=48)	Primary: MTD; Secondary: CR, best overall response rate, PFS, OS, acute, and chronic GvHD	July 2021
Experimental: Decitabine + Talacotuzumab (anti-CD123 mAb) Active comparator: Decitabine	Phase II/III; NCT02472145	AML suitable for experimental therapy or not eligible for intense induction chemotherapy (n=326)	Primary: CR, OS; Secondary: EFS; proportion of patients with CR with MRD and negative CRi; time to best response; duration of response	January 2018
Experimental: Vadastuximab talirine (anti-CD33 mAb) + Azacitidine or Decitabine Active comparator: Placebo + Azacitidine or Decitabine	Phase III; NCT02785900	Adult patients with newly-diagnosed AML (n=240)	Primary: OS, composite complete remission (CRc) rate; Secondary: MRD, duration of remission, survival, AEs, abnormalities, mortality rates	October 2017

AE, Adverse event; AUC, Area under the curve; C_max_, Peak concentration that a drug achieves in a specified compartment after the drug has been administrated and before a second dose administration; CR, Complete remission; CRi, CR with incomplete blood cells count recovery; CRp, Complete remission with incomplete platelet recovery; EFS, Event-free survival; GvHD, Graft-versus-host disease; MRD, Minimal residual disease; MTD, Maximum tolerated dose; OS, Overall survival; PFS, Progression-free survival; T_max_, Amount of time that a drug is present at the maximum concentration in serum. *Results have not been reported.

T-cell immunoreceptor with Ig and ITIM domains (TIGIT) is a novel coinhibitory receptor expressed by T and NK cells that binds to CD155, leading to impaired T or NK cell anti-tumorigenic functions (98). TIGIT is an emerging target in cancer immunotherapy where its activation in T cells by its ligand CD155 or CD112 expressed by cancer cells inhibits T cell responses (99). Interestingly, AML mice model harboring CD155-specific deletion (murine AML cells TIB-49) showed prolonged survival upon administration of decitabine and DC/AML fusion vaccine compared with either agent alone (100). A recent study that profiled the expression of specific immune cells and immune checkpoint markers in peripheral blood of 14 elderly AML patients treated with decitabine (pre- versus post-treatment, and in responders vs non-responders) suggested that combination of decitabine with novel ICB therapy such as anti-TIGIT antibodies might be a better therapeutic strategy than with conventional ICB regimens (anti-PD-1/L1 or -CTLA-4 antibodies) (101). In AML patients before and after initiation treatment with decitabine, no significant changes were observed for all immune cell populations post-decitabine treatment including CD4^+^ or CD8^+^ T cells, Tregs, NK cells, NKT cells, B cells, DCs, and MDSCs. PD-1 expression was not altered upon decitabine treatment, and no significant difference in its expression was observed between responders and non-responders to decitabine. Nonetheless, stimulated CTLs from responders produced significantly higher IFN-γ than non-responders, while non-responders showed higher expression of multiple immune checkpoints including TIGIT in CTLs and NK cells, and CD38 in CTLs and CD4^+^ cells (101). These suggest that combination of decitabine with ICB therapy targeting TIGIT or CD38, instead of PD-1/L1 and CTLA-4, might yield better clinical outcomes.

## Activation of Antileukemic NK Cells

NK cells play vital roles in cancer immunosurveillance that can directly target and destroy cancer cells. Upon recognition, NK cells form immunological synapse with cancer cells, causing specific lysis of target cells through the release of tumor necrosis factors (TNFs), death-inducing ligands such as FAS ligand, and TNF-related apoptosis-inducing ligand (TRAIL) present on the surface of NK cells. Binding of these ligands by cancer cells (*e.g.* through the FAS receptor) leads to apoptosis (102, 103). Various stimulatory and inhibitory receptors are expressed by NK cells that orchestrate NK cell activities. Natural killer group 2D (NKG2D, also known as CD314 and encoded by the gene *KLRK1*) is the best characterized NK cell activating receptor that recognizes tumor cells that express NKG2D ligands. A group of eight NKG2D ligands (NKG2DLs) have been identified comprising of MHC class I polypeptide-related sequence A (MICA), MICB, and the UL16-binding proteins (ULBP) 1–6 (103). One of the immune escape mechanisms in AML is the suppression of NK cell antileukemic activities.

In terms of normal NK cells derived from healthy human donors, their proliferation (induced by K562-based artificial antigen presenting cells Clone9.mbIL21) and viability were reduced by decitabine treatment in a dose-dependent manner (0.02–20 µM) (104). This was in line with dose-dependent increased and reduced expression of KIR and NKG2D, respectively. Moreover, DNA hypomethylation increased following decitabine treatment at concentrations up to 0.3 µM, but methylation returned to baseline levels beyond that concentration, suggesting that low-dose decitabine is required to activate NK cells while high-dose conferred the opposite effect.

NK cells produced from CD34^+^ hematopoietic stem and progenitor cells (HSPC-NK cells), and subsequently treated with low-dose decitabine preserved their proliferation and IFN-γ production capacity but azacitidine attenuated such effects. In NOD/SCID/IL2Rg^null^ mice, decitabine but not azacitidine was capable of potentiating infused HSPC-NK cells’ antileukemia activity through upregulation of ligands in AML cells (THP-1) for NKG2D and DNAX accessory molecule-1 (DNAM-1) immunoactivating receptors on HSPC-NK cells. Moreover, decitabine increased the proliferation of HSPC-NK cells in their bone marrow, and combination therapy of adoptive HSPC-NK cells with decitabine was proposed for treatment of AML patients (105). The study was in line with another study on decitabine treatment in a mouse ovarian cancer model (BR5FVB1-Akt cells in FVB mice) where low-dose decitabine increased chemokines expression that recruited NK cells and CTLs, and promoted their production of IFN-γ and TNF-α (106). The authors proposed that a 10-day decitabine treatment might be more efficacious than the 5-day treatment performed in their mice study.

In AML blasts, CD33 is frequently expressed and it represents a therapeutic target for the disease. BI 836858 is an Fc-engineered anti-CD33 therapeutic antibody that activates autologous and allogeneic NK cell-mediated antibody-dependent cellular cytotoxicity (ADCC) in AML cells with opsonized BI 836858. In serial marrow aspirates of elderly AML patients receiving decitabine, BI 836858-mediated ADCC was enhanced when compared with pre-decitabine treatment. This was mediated by increased mRNA expression of ligands to the activating receptor NKG2D, and blocking of NKG2DL receptor decreased BI 836858-mediated ADCC (107). Limitations of the study, as noted by the authors, include lack of serum NKG2DL measurement post-decitabine treatment as AML blasts could shed NKG2DL to escape immune surveillance, as well as lack of autologous NK cells and AML blasts for measurement of surface NKG2DL protein levels in patients treated with decitabine.

Adoptive NK cells transfer is a promising therapeutic strategy for hematologic malignancies. In a recent phase I study (NCT02316964) reported this year, decitabine treatment followed by haploidentical NK cells infusion and IL-2 (aldesleukin) administration in R/R AML patients demonstrated that no donor-derived NK cells were detected post-NK cell infusion (time points tested: 2, 8, 14, 21, and 28 days post-NK cell infusion) (108). The protocol was thus amended to include the chemotherapy drug fludarabine, and short-term (up to 2 days) engraftment of donor-derived NK cells were detected without infusional toxicities in the NK cells. It was concluded that decitabine and NK cells infusion regimen was safe, but decitabine combination with fludarabine and IL-2 was insufficient to maintain persistence of the infused donor NK cells *in vivo*. The authors subsequently generated membrane-bound IL-21 (mbIL-21) NK cells from normal donors expanded with mbIL-21^+^ K562 AML cells. The *ex vivo* expanded mbIL-21 NK cells showed effective lysis of primary AML blasts (derived from the AML patients enrolled in the clinical trial) *in vitro* and *in vivo* (patient-derived xenograft mice) which was further enhanced by Fc-engineered anti-CD33 mAb combination (108).

In other types of blood cancer, decitabine also induced NK cell-mediated anti-tumor immunity through increased IFN-γ production against CML (K562) and Burkitt’s lymphoma (Raji) cells (109). Azacitidine was reported to induce NK cells apoptosis and impaired mRNA synthesis but, in contrast, decitabine enhanced the activation of NK cells by inducing transcription of NK reactivity genes (109). Overall, translation of decitabine-mediated NK cell activation therapeutic approach into actual patients remains to be determined including the appropriate dosage (*e.g.* low-dose decitabine) and cycles of decitabine required to achieve similar antileukemic effects by human NK cells. It is also unclear if low-dose decitabine also similarly activates T cell anti-tumor responses.

## Suppression of Pro-Leukemic Myeloid-Derived Suppressor Cells

MDSCs are a group of heterogenous immature myeloid cells mostly consisting of CD33^+^/CD11b^+^/HLA-DR^-^ cells that form an immunosuppressive niche in cancers. MDSCs suppress anti-tumor responses through multiple mechanisms including depletion of amino acids required for T cell proliferation (110), generation of reactive oxygen species (ROS) that alters T cell receptor and CD8 molecules leading to T cell tolerance (111), antigen presentation to induce Treg cells activities, and the production of anti-inflammatory cytokines such as TGF-β and IL-10 (112). In AML patients, the frequency of MDSCs is increased in the peripheral blood and bone marrow associated with minimal residual disease (113, 114). Chemo-immunotherapy combination regimen improved the survival of AML mice model (C1498 murine AML cells in C57Bl/6J mice) along with stable reduction in MDSC populations (76). In addition, administration of anti-CD33/CD3 antibody (AMG 330) triggered T cell-mediated lysis of AML blasts that was further enhanced by T cell elimination of IDO^+^CD33^+^ MDSCs (IDO, an enzyme released by cancer cells to deplete tryptophan leading to suppressed T cell activities) (115).

Decitabine treatment depleted MDSC populations (Gr1^+^/CD11b^+^) in normal (BALB/c), mouse AML (WEHI-3 cells in BALB/c) and lymphoma (EL4 cells in C57/BL6) mice models with minimal changes to other immune effector cells (CD4^+^ T cells, CD8^+^ T cells, NK cells, B cells, Tregs, and DCs). The compound induced apoptosis of MDSCs from normal BALB/c mice, and activated CD4^+^ and CD8^+^ T cell responses in the mice bone marrow through depletion of MDSC populations. More importantly, in the same study, an adoptive transfusion mouse model demonstrated that decitabine treatment was capable of inducing autologous T cell responses against leukemia cells *in vivo* (decitabine-treated WEHI-3 cells in BALB/c nude mice) by depleting MDSCs (116). One of the limitations of the study was that the mechanism involved in the apoptosis induction of MDSCs by decitabine remained unknown, and it was thought to be due to cell cycle arrest and inhibition of the STAT3/STAT5 signaling pathway crucial for MDSCs differentiation. Furthermore, MDSCs have been shown to restrain NK cell-mediated killing of myeloid cancers, and depletion of MDSCs restores NK cell cytolytic function in MDS (117–119). It is of future interest to investigate if decitabine-mediated depletion of MDSCs also leads to activation of antileukemic NK cells.

Apart from AML, the efficacy of decitabine in the inhibition of MDSCs has also been reported in multiple myeloma (MM) which remains an incurable malignancy that requires novel therapies. Transwell co-culture of mouse IL-6-secreting MM cell line MPC11 with MDSCs showed reduction in IL-6 production by mouse MM cell line (MPC11) and increased apoptosis occurred when the MDSCs were derived from decitabine-treated bone marrow cells compared with the control-treated (PBS) counterpart (120). These decitabine-mediated inhibitory effects were rescued when supplemented with monocytic MDSCs (M-MDSCs). It was also reported that in MPC11-bearing mice (BALB/c), combination of decitabine with anti-Gr1 antibody (mouse MDSCs express high levels of the granulocytic marker Gr1) demonstrated synergistic effects against tumor growth. This was accompanied by depleted M-MDSCs and increased proliferation of T cells in the tumor microenvironment, and these effects were rescued by M-MDSC reinfusion (120). Hence, decitabine enhanced autologous T cell responses by depleting M-MDSCs in the microenvironment of MM.

MDSCs are classified into two major groups: M-MDSCs and granulocytic MDSCs (G-MDSCs) where each group morphologically and phenotypically resembles monocytes and neutrophils, respectively, and M-MDSCs confer stronger immunosupressive activities than G-MDSCs (121, 122). It may be of therapeutic interest to examine the potential differential effects of decitabine and guadecitabine against each MDSCs subpopulation as both M-MDSCs and G-MDSCs demonstrate relatively different profiles in AML and MDS. For instance, depletion of M-MDSCs was relatively higher than G-MDSCs depletion in AML mouse model (C1498 in C57Bl/6J mice) treated with cytosine arabinoside (AraC) monotherapy or combination of AraC with Plerixafor (an immunostimulant to direct HSCs into peripheral circulation) and anti-PD-L1 mAb (76), while higher M-MDSCs frequency was observed in both low- and high-risk MDS compared with normal individuals (123).

## Synergism with Anti-CD123 Therapeutic Antibodies

Interleukin-3 receptor (IL-3R) is a heterodimeric receptor comprising of the IL-3-specific alpha subunit (also known as CD123 or IL-3RA) and the beta subunit (CD131 or IL-3RB) that is shared by receptors for IL-5 and granulocyte-macrophage colony-stimulating factor (124). IL-3 initially binds to CD123 that subsequently recruits CD131 to form the high-affinity IL-3R receptor, resulting in activation of the JAK/STAT pathway to produce anti-apoptotic proteins crucial for hematopoietic cell viability (125, 126). CD123 expression is typically very low or absent in normal hematopoietic cells but it is broadly expressed in numerous hematological malignancies including MDS and AML where it is expressed in over 90% of AML cases at various intensities (127). CD123 overexpression in AML blasts and LSCs is associated with higher blast counts at diagnosis, poorer CR, and survival (127, 128), thereby establishing CD123 as a promising therapeutic target in AML.

Tagraxofusp (SL-401 or DT_388_IL-3) is an engineered fusion protein comprising of IL-3 to target CD123 fused with a truncated diphtheria toxin (DT) payload. Tagraxofusp triggers cellular cytotoxicity by delivering DT to CD123^+^ cells where DT escapes endosomes post-internalization and catalyzes ADP ribosylation of eukaryotic elongation factor 2 (eEF2; required for the translocation step in protein synthesis), leading to inhibition of protein synthesis that kills the cell (129). In 2018, tagraxofusp was the first approved therapy specifically for blastic plasmacytoid dendritic cell neoplasm (BPDCN), a rare and aggressive hematological malignancy that highly expresses CD123 (130). Tagraxofusp monotherapy in AML patients had been tested in phase I trial where it was safe and dose levels of ≥3.0 mg/kg was recommended to maximize ADCC against residual AML cells (131). Another phase I/II trial is ongoing to assess tagraxofusp monotherapy in AML patients in first CR or with MRD-positive disease where interim results demonstrated that no dose-limiting toxicities or maximum tolerated dose (MTD) was observed in the highest tested dose of 12 µg/kg (NCT02270463) (132).

Resistance to tagraxofusp has been observed in some BPDCN patients while others relapse after exhibiting a clinical response. In experimental studies, it was recently shown that BPDCN and AML cells resistant to tagraxofusp treatment were not associated with CD123 loss but rather due to deficiencies in the diphthamide synthesis pathway (133). Specifically, this was due to loss of DPH1 expression, an enzyme involved in diphthamide synthesis, in BPDCN and AML primary patients resistant to tagraxofusp treatment. Tagraxofusp-resistant AML cells (THP1) displayed CpG motifs hypermethylation in the promoter region of *DPH1*, and azacitidine treatment suppressed the CpG DNA hypermethylation that restored *DPH1* expression. In the same study, combination of tagraxofusp and azacitidine was effective against primary human leukemias *in vivo* (recipient mice injected with BPDCN patient-derived xenograft cells) where their combination prolonged survival of the mice compared with either agent alone (133). Phase I study of tagraxofusp in combination with azacitidine in R/R AML and high-risk MDS patients is currently underway to determine the MTD and response rates (NCT03113643). These results are eagerly awaited as HMAs may enhance the efficacy of anti-CD123 immunotherapy in R/R AML and MDS.

Talacotuzumab (JNJ-56022473, formerly CSL362) is another therapeutic mAb against CD123 where it is a humanized, affinity-matured and Fc-engineered mAb for increased affinity to CD16 expressed by innate effector cells. Talacotuzumab potently induces AML patient’s own NK cells to destroy AML blasts and LSC-enriched populations *via* ADCC (134, 135). Phase I trial demonstrated that talacotuzumab was well-tolerated in AML patients (n=24) with high risk of relapse and potent ADCC against residual AML was observed (131). However, recent (2020) trials have independently reported significant toxicities of talacotuzumab treatment in AML patients. In a pivotal phase II/III study (NCT02472145; the SAMBA trial) of talacotuzumab in combination with decitabine as first line treatment in elderly (65 years or older) patients with *de novo* or secondary AML not eligible for intensive chemotherapy, the combination lacked in efficacy and with high toxicity rates leading to premature termination of the trial and discontinuation of talacotuzumab treatment (136). An independent phase II trial of high-risk MDS (n=5) and AML (n=19) patients resistant to previous HMAs (azacitidine or decitabine) treatment showed that single agent talacotuzumab conferred limited efficacy but significant toxicities such as infections, cytopenias, cardiac, gastrointestinal and nervous system disorders, resulting in high number of patients with treatment discontinuation (128). The authors suggested that utilization of other immune modalities such as CD123-specific chimeric antigen receptor-engineered T cell (CAR-T) therapy may improve the unfavorable risk/benefit profile of talacotuzumab therapy.

In terms of bispecific antibodies (bsAbs), the following bsAbs targeting both CD123 and CD3 (CD123 x CD3 bsAb) with promising efficacy are currently being assessed in early phase clinical trials of R/R AML patients, and their combination with HMAs has yet to be investigated: i) Flotetuzumab (MGD006 or S80880) is a bispecific dual-affinity re-targeting antibody (DART) that recognizes CD123 and CD3ϵ where it redirects T cells to destroy CD123-expressing cells. It was granted orphan drug designation by FDA for the treatment of AML in January 2017 (137). In R/R AML patients with *TP53* mutations, 47% (n=7/15) demonstrated CR to flotetuzumab and displayed increased tumor inflammation signature as well as CD8, inflammatory chemokine, and PD-1 expression compared with non-responders. These patients who achieved CR experienced prolonged survival (138). Phase I/II trial is being evaluated in R/R AML and high-risk MDS patients (NCT02152956) to further assess the efficacy of flotetuzumab therapy; (ii) XmAb14045 (SQZ622) is another CD123 x CD3 bsAb. In contrast to smaller antibody constructs such as DARTs, XmAb14045 is a full-length immunoglobulin molecule with a unique Fc domain in which binding with Fcγ receptor (FcγR; broadly expressed by both lymphoid and myeloid cells) is abolished to prevent non-selective T cell activation but with preserved neonatal Fc receptor (FcRn) binding to maintain long serum half-life. XmAb14045 has shown efficacy against CD123^+^ AML cells (139). Current phase I study is evaluating its safety, MTD and recommended dose in various hematological malignancies including R/R AML (NCT02730312).

## Synergism with Chimeric Antigen Receptor-Engineered-T (CAR-T) and T Cell Receptor-T (TCR-T) Cells

CAR-T cells have shown promises in the treatment of hematological cancers but their exhaustion after infusion in patients limits clinical efficacy. In experimental settings, *de novo* DNA methylation mediated by DNMT3A is one of the key mechanisms that restrains long-term T cell memory and induces cytotoxic T cells exhaustion (85, 140). Essentially, recent experimental study demonstrated that decitabine significantly enhanced anti-AML effects of CD123 CAR-T cells *in vitro* (THP1 cells) and *in vivo* (NSG mice bearing THP1 tumor xenografts). This was achieved by decitabine through inhibition of DNMT3A and DNMT1 expression, increased DNA hypomethylation and upregulated expression of genes that favored naïve and memory T cells differentiation, resulting in enhanced CD123 CAR-T cells anti-tumor responses (141). This is consistent with past observations that naïve and memory T cell populations are superior to effector T cells in triggering anti-cancer effects for adoptive cell therapy (142). As pre-clinical study has showcased the potential of HMAs combined with CD123 CAR-T-based immunotherapy in AML patients, translation to actual clinical trials is thus desirable.

Recent progress has been made to generate NKG2D CAR-T cells by fusing the full-length human NKG2D to the human CD3ζ cytoplasmic signaling domain for autologous adoptive cell therapy in AML patients. These efforts have recently been tested in phase I trials in which functional activity of NKG2D CAR-T cells against NKG2DL-positive cells were achieved in AML patients without significant toxicities, although no objective tumor responses were observed (143, 144). Further clinical trial modifications are required still to determine the optimal dose of NKG2D CAR-T cells in AML patients, and whether combination with HMAs could augment NKG2D CAR-T cells expansion or upregulation of NKG2DLs expression in AML cells for NKG2D CAR-T cells recognition is an open question.

Apart from CAR-T therapies, the specificity of T cells can be redirected toward selected tumor antigens by transduction with an exogenous T cell receptor (TCR) targeting the specific antigen, and this forms the basis of TCR-engineered T cell (TCR-T) therapy (145, 146). TCR-T cells specifically targeting Wilms’ tumor 1 (WT1; overexpressed in AML and MDS cells) demonstrated HLA-A*24:02-restricted cytotoxicity against WT1-expressing myeloid leukemias (147). In phase I trial of refractory AML and high-risk MDS patients (n=8), adoptive transfer of WT1-specific TCR-T cells did not confer adverse events and the TCR-T cells persisted throughout the study period with retained WT1-specific immune reactivity in majority of the patients (148). In a therapy-related MDS patient who had relapsed after allo-HSCT, treatment with azacitidine combined with DLI yielded a strong GvL effect along with WT1-specific CD8^+^ T cell responses that resulted in remission for 15 months before the patient finally relapsed (149). The GvL effect and prolonged memory phenotype of the WT1-specific CTL was induced at least partially by azacitidine treatment, and this supports the potential synergism of decitabine or guadecitabine with TCR-T therapy.

## Conclusions and Future Directions

**Figure 2:**
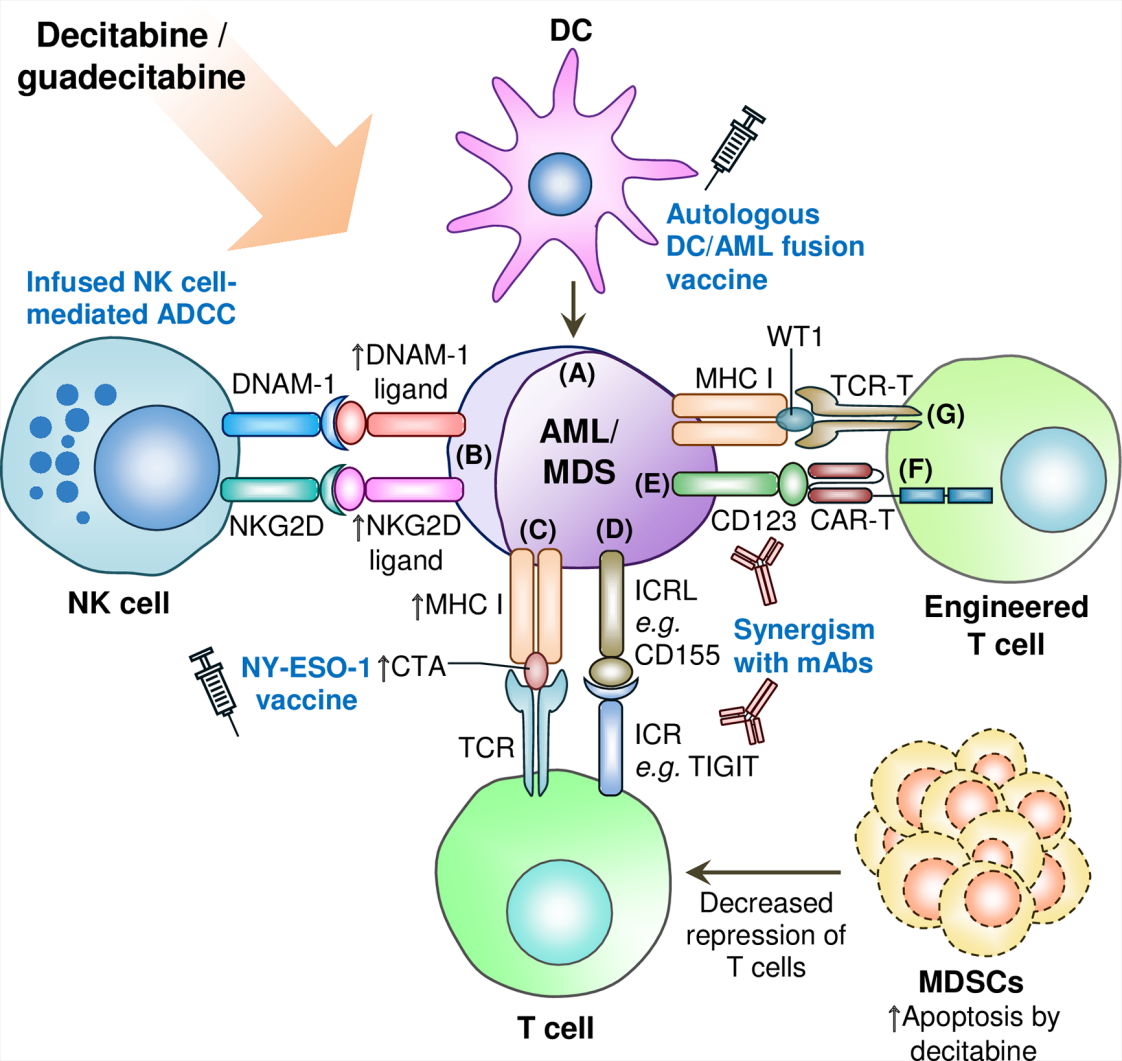
Synergism of decitabine or guadecitabine with antileikemic immune cells and engineered T cells, and depletion of immunosuppressive cells in AML or MDS microenviroment. **(A)** DC/AML vacinne combined with decitabine or guadecitabine treatment induces leukemia-specific immunity; **(B)** Decitabine potentiates infused NK cells’ antileukemia activity by upregulating ligands for NKG2D and DNAM-1 immunoactivating receptors on NK cells. Decitabine combined with anti-CD33 mAb also augments the expression of NKG2D ligands in AML cells; **(C)** Upregulation of CTA expression such as NY-ESO-1 by decitabine or guadecitabine treatment synergizes with NY-ESO-1 cancer vaccine treatment; **(D)** Combination of decitabine or guadecitabine with novel immune checkpoint blockade therapy such as anti-TIGIT therapeutic antibody to circumvent resitance of AML or MDS cells to either hypomethylating agent; **(E)** Potential synergsim of decitabine or guadecitabine with anti-CD123 therapeutic antibody (*e.g.* tagraxofusp) to induce antileukemic immunity; **(F)** Decitabine treatment synergizes with CD123 CAR-T cells against AML cells; **(G)** Potential synergism of decitabine or guadecitabine with WT-1 specific TCR-T cells. ADCC: Antibody-dependent cellular cytotoxicity; AML: Acute myeloid leukemia; CAR-T: Chimeric antigen receptor-engineered T cell; CTA: Cancer/testis antigen; DC: Dendritic cell; DNAM-1: DNAX accessory molecule-1; ICR: Immune checkpoint receptor; ICRL: Immune checkpoint receptor ligand; mAb: Monoclonal antibody; MDS: Myelodysplastic syndromes; MDSC: Myeloid-derived suppressor cell; MHC: Major histocompatibility complex; NK: Natural killer; NKG2D: Natural killer group 2D; TCR: T cell receptor; TCR-T: T cell receptor-engineered T cell; TIGIT: T-cell immunoreceptor with Ig and ITIM domains; WT1: Wilms’ tumor 1. Upward arrow denotes upregulated expression, increased CTA presentation, or induced apoptosis of MDSCs.

In conclusion, decitabine and guadecitabine have shown promising immunopotentiating properties to improve the efficacy of immunotherapies (**Figure 2**). In particular, early clinical trial of decitabine combination with NY-ESO-1 vaccine (anti-DEC-205-NY-ESO-1 fusion protein and poly-ICLC) has yielded favorable results of consistent NY-ESO-1 expression upregulation to stimulate leukemia-directed T cell immune responses. Another early phase clinical trial (NCT03358719) is currently underway to investigate combination of decitabine with the NY-ESO-1 vaccine and nivolumab (anti-PD-1 mAb) in AML and MDS patients. Decitabine capabilities to augment both NY-ESO-1 and PD-1 expression in leukemia cells may prove as a strong adjunct to prime the patients to NY-ESO-1 vaccine as well as anti-PD-1 mAb regimen. In addition, guadecitabine combination with atezolizumab in R/R AML patients where the primary outcomes measured include clinical responses such as CR has recently been completed and reporting of results are eagerly awaited (NCT02892318; **Table 2**).

Regarding future directions, combination of decitabine or guadecitabine with antibodies targeting novel T cell inhibitory receptors such as anti-TIGIT mAb may constitute the optimal ICB therapy combination with the HMAs, and whether such combination also enhances the clinical responses of DC/AML fusion vaccine should be examined. In terms of adoptive cell therapy, the translational implications of decitabine or guadecitabine synergism with infusion of donor-derived NK cells, CD123/NKG2D CAR-T or WT1 TCR-T cells targeting AML and MDS cells represent fertile areas for future investigations. Finally, combination of these HMAs with anti-tumor immunomodulatory inhibitors (150–153) including small molecule immunomodulatory drugs (*i.e.* thalidomide, lenalidomide and pomalidomide) shown to confer antileukemic T cell immunity in AML and MDS (154–156) represent exemplary avenues for further explorations to achieve and maintain a robust antileukemic milieu in this complex group of blood malignancies.

## Author Contributions

KKW conceived and designed the manuscript, prepared the figures and tables, and wrote and revised the manuscript. NSY and RH critically edited and revised the manuscript. All authors contributed to the article and approved the submitted version.

## Funding

This work was supported by the Research University Grant (1001/PPSP/8012349), Universiti Sains Malaysia awarded to KKW.

## Conflict of Interest

The authors declare that the research was conducted in the absence of any commercial or financial relationships that could be construed as a potential conflict of interest.
